# Pennsylvania Hospital

**Published:** 1839-01-05

**Authors:** 


### CLINICAL LECTURES.

### PENNSYLVANIA HOSPITAL.

### EXTIRPATION OF THE PAROTID GLAND.

*Wednesday*, 19*th December*.—Dr. Randolph entered the amphitheatre, and commenced as follows:—

*Gentlemen*,—We propose to perform an operation to-day, for the removal of a tumour, which we believe to involve the parotid gland. Inasmuch as this operation must necessarily be a tedious one, and consume a good deal of time, I shall detain you but for a few moments, in order to give you an account of the case. I have said to you, gentlemen, that we *believe* this tumour to involve the substance of the parotid gland. Upon this point, however, we do not give a positive opinion, because it sometimes happens that tumours form in the substance of the parotid gland, in consequence of an enlargement of a lymphatic gland, and attain some size, and yet admit of removal, leaving behind the parotid itself. At other times, a tumour may form exterior to the parotid, and attain a large size, and, by its compression upon the parotid, cause its partial absorption, so as to seem to occupy its precise situation. Judging from the feel and external appearance of this morbid growth, I am of opinion that it involves the parotid gland; still, for the reasons I have just given, we cannot decide this point positively, until after the operation.

The patient is a coloured man, about fifty years of age, an inhabitant of the southern part of the state of Delaware. The history which he gives of his case, is this:—He first perceived, about twelve months ago, a small tumour of the size of a marble, just anterior to the lobe of the left ear. The tumour at that time was moveable, and did not give him much pain; it continued to increase in size, and some months after he first noticed it, he showed it to a highly respectable medical gentleman, living in his neighbourhood, who advised its removal. The operation, however, was not performed, and the tumour has gone on increasing gradually in size up to the present time. He came to this city, and, through the politeness of Dr. Beasly, was placed under my charge. Since his admission into the hospital, ten days ago, the tumour has sensibly increased, according to the observation of Dr. Smith, myself, and other persons connected with the institution, and is now about the size of an orange. Now, as far the operation is concerned, I would much prefer that the tumour had been stationary for some time, because, in that case, I might infer that the hardness and weight of the tumour would, by its compression, have obliterated some of the vessels supplying it with blood, so as to diminish the hæmorrhage attending its removal. Inasmuch, however, as the tumour is still enlarging, it is probable that its blood-vessels are increased in size, so as to furnish it with a supply of blood adequate to its growth, and we may expect to find it in a highly vascular condition.

You are aware, gentlemen, that doubts have been expressed, whether the parotid gland has ever been completely and successfully extirpated from the living subject. The limits of my time will not permit me to enter into a prolonged argument upon this subject; but I must content myself with the simple statement, that to deny the fact of the extirpation of the parotid gland, is, in my opinion, to deny the testimony of some of the most respectable and distinguished authorities in anatomy and surgery. We have accounts of the extirpation of the parotid gland, which may be dated at least as far back as a century ago. Heister relates a case in which a diseased parotid gland was extirpated in the year 1733. Subsequently, several cases are reported by the older surgeons, in which the operation had been performed. I may mention the names of Siebold, Sourcrampe, Scultetus, Gooch, and Behr, all of whom have reported cases. Mr. Bell, in his "Essay on Tumours," states that he removed the parotid gland. More recently, Mr. Abernethy, in the seventh volume of the Medico-Chirurgical Transactions, relates a case in which Mr. Goodland, of Bury, removed the whole of the parotid gland, in September, 1815. In 1818, it was successfully removed by Mr. Carmichael, a surgeon of Dublin. A case is reported in 1824, in which Beclard, the late distinguished Professor of Anatomy in the "Ecole de Médicine," at Paris, asserts that the parotid gland was entirely removed: this patient died on the 15th day after the operation, from erysipelatous inflammation, and, upon dissection, it was satisfactorily proved that every particle of the gland was removed. Besides these cases, the operation has been performed in Europe by Gensoul of Lyons, Lisfranc of Paris, Sir Astley Cooper, and some others.

The diseased parotid gland was first successfully extirpated in this country by Professor George M'Clellan, of this city, in 1826:* since that time, he has twice removed it. The operation has also been performed by Professor Mott, and the late Dr. Bushe, of New York, by the late Professors Davidge and Smith, of Baltimore, and by Professors Dudley and N. R. Smith, of Lexington, Kentucky.

In the London Medical Gazette, for June last, a case is communicated by Sir James M'Grigor, in which Mr. Widmer, surgeon to the British forces at Toronto, Upper Canada, successfully extirpated this gland. Now, gentlemen, admitting that in some of these cases mistakes have been made, and that tumours resembling the parotid have been taken out, and not the gland itself, still, this objection cannot surely apply to *all* the cases; for we cannot suppose that the most learned and distinguished anatomists and surgeons in the world would be so ignorant as not to know the parotid gland when they saw it removed from its natural situation, and the place which it ought to occupy left empty and void.

I have been in the habit of stating in my lectures, for a number of years, that I believe in many instances a diseased parotid gland could be more easily removed than the natural one, for this reason: parts, in a state of tumefaction and scirrhous enlargement, frequently, by their compression, produce a partial absorption of the adjacent tissues; besides which, one of the characteristics of scirrhus, is to contract itself, so that the tumour isolates itself, in a measure, from its former connexions. I was led to this belief, by noticing the facility and quickness, with which I have been able, in some instances, to extirpate a scirrhous mammary gland, which, in its natural condition, would necessarily occupy a good deal of time, and be attended with some difficulty. In two or three instances, in performing this operation, after the first incisions, I have been able to raise up the diseased gland, and, with a very few strokes of the scalpel, separate it from its loose cellular connexions. In the report of the operation for the removal of the parotid gland, by Dr. N. R. Smith, I see that he expresses nearly the same sentiments.

I will now briefly state the manner in which I propose performing the operation. I shall make an incision commencing at the zygoma, and continue it down over the surface of the tumour, until it reaches the edge of the sterno-mastoid muscle,—and then a second incision, intersecting this at right angles. I shall next raise up the flaps, and dissect up the tumour from below. My object in doing this, is to cut down for the external carotid artery, and endeavour to take it up just before it enters the gland, in the space between the edge of the stylo-hyoid muscle and the angle of the lower jaw. It is possible, however, as the tumour extends so low down, that it may be necessary to take up the common trunk of the carotid. In removing the tumour from its bed, between the mastoid process and the angle of the jaw, I shall use, as much as possible, the handle of the scalpel.

[Dr. Randolph then proceeded to operate, in the presence of Drs. Coates, T. Harris, J. Rhea Barton, Norris, and Horner, besides several other highly respectable physicians and surgeons, and an unusually numerous class of students.

The patient was placed upon a table, his head inclined to the right side, and an incision made from just above the zygoma downward and forward over the whole extent of the tumour, to the edge of the sterno-mastoid muscle. A second incision was then made, which intersected this at right angles. Upon dissecting up the flaps, and raising the lower edge of the tumour, the facial artery was exposed, and it was judged advisable to secure it with a ligature. The adhesions were found to be so strong and close, that it was necessary to dissect the whole mass from its situation with the edge of the knife, as no separation could be effected with the handle of the scalpel. The tumour adhered so very firmly at its lower part to the angle of the jaw, and projected so far beyond it, that it was found impracticable to secure the external carotid artery at the place contemplated, and it was thought that the

* It is stated in Cooper's Surgical Dictionary, (American edition,) by Dr. Reese, the editor, that the parotid gland was extirpated by Dr. White, of Hudson, in 1808.—*Eds.*

diseased mass could be more readily removed by separating it above, and then drawing it out. The dissection was accordingly continued from above downward, and, in doing this, it became necessary to secure the temporal and internal maxillary arteries, besides several others of smaller size, which bled with great freedom. The external jugular vein was also cut, and secured at both ends. In dividing the last bands which confined the tumour to its situation, the external carotid artery was cut, and poured out a large stream of blood; it was immediately secured with a ligature thrown round the artery by means of Dr. Physick's needle and forceps. A portion of the masseter muscle was so firmly attached to the diseased mass, as to render the removal of it necessary. The periosteum, also, covering a portion of the angle of the lower jaw, was absorbed, and the tumour adhered to the bone itself. The whole gland was finally separated quite unbroken, and, when removed, the bare styloid process and the stylo-maxillary ligament were distinctly seen.

In consequence of the division of the portio dura, the operation was necessarily followed by paralysis of the muscles on that side of the face. It should be stated, however, that paralysis existed previous to the operation; and it is somewhat singular that it was not augmented by the division of the nerve.

The wound was closed by a few stitches of the interrupted suture, and a light dressing applied. The operation lasted fifty-nine minutes.

Upon a careful examination of the parts, after the operation, it was unanimously declared by the surgeons and anatomists present, that *the whole of the parotid gland was completely removed.*]

*Case continued.*—Evening. Patient complained but little; slight oozing from the wound; no fever; pulse good. Ordered gum water, sago, and fifty drops of laudanum.

*20th.* Has slept well; little pain; pulse eighty-four, full, and regular. Diet continued; an enema. *Evening.* Pulse full, slightly accelerated; no headache, or other bad symptom. Ten grains of Dover's powder.

*21st.* Has slept well; slight cough, which is in some degree kept up by the tightness of the bandage on the throat. Bowels opened by an injection this morning; pulse regular, eighty-four; tongue moist, slightly furred. Expression of countenance and spirits good. Ordered the soft parts of six stewed oysters; milk at night; soda biscuits.

*23d.* Wound dressed to-day; suppurating; slight inflammation around the parts. Cough better; pulse natural; tongue moist. Dressings renewed; diet continued.

*24th.* Up to this date, five days after the operation, no bad symptom; to-day, slight erysipelas on face and ear; a little fever; pulse ninety-six, but regular. Wound suppurating. Poultice to wound, and soap liniment rubbed over the side of the face. Bowels opened by injection; Dover's powder.

*25th.* Symptoms good; three sutures removed; treatment continued.

*26th.* Erysipelas disappearing and wound suppurating freely and granulating; swelling of the face disappearing. One small ligature came away.

*27th.* Erysipelas nearly gone; other symptoms favourable; cerate to wound.

*29th.* Still improving. No paralysis except at the angle of the mouth. Two ligatures came away. Mucilage to wound.

*31st.* Wound healed at the corners; elsewhere granulating freely; parts perfectly healthy. A large ligature came away. Sits up in bed.

*2d.* Wound almost healed. General health excellent.

